# Molecular Characterization and Phylogenetic analyses of Rotaviruses Circulating in Municipal Sewage and Sewage-Polluted River Waters in Durban Area, South Africa

**DOI:** 10.1007/s12560-024-09598-z

**Published:** 2024-06-24

**Authors:** Cornelius Arome Omatola, Ademola Olufolahan Olaniran

**Affiliations:** Discipline of Microbiology, School of Life Sciences, College of Agriculture, Engineering and Science, University of KwaZulu-Natal, Westville Campus, Private Bag X54001, Durban, 4000 Republic of South Africa

**Keywords:** Rotavirus, Genotypes, Molecular diversity, Sewage, River, South Africa

## Abstract

Globally, rotavirus continues to be the leading etiology of severe pediatric gastroenteritis, and transmission of the disease via environmental reservoirs has become an emerging concern in developing countries. From August to October 2021, a total of 69 samples comprising 48 of raw and treated sewage, and 21 surface waters, were collected from four Durban wastewater treatment plants (DWWTP), and effluent receiving rivers, respectively. Rotaviruses recovered and identified from the samples were subjected to sequencing, genotyping, and phylogenetic analysis. Of the 65 (94.2%) rotavirus-positive samples, 33.3% were from raw sewage, 16% from activated sludge, 15.9% from final effluents, and 29.0% were from the receiving river samples. A total of 49 G and 41 P genotypes were detected in sewage while 15 G and 22 P genotypes were detected in river samples. G1 genotype predominated in sewage (24.5%) followed by G3 (22.4%), G2 (14.3%), G4 (12.2%), G12 (10.2%), G9 (8.2%), and G8 (6.1%). Similarly, G1 predominated in river water samples (33.3%) and was followed by G2, G4 (20.0% each), G3, and G12 (13.3% each). Rotavirus VP4 genotypes P[4], P[6], and P[8] accounted for 36.6%, 29.3%, and 9.8%, respectively, in sewage. Correspondingly, 45.5%, 31.8%, and 13.6% were detected in river samples. The G and P genotypes not identified by the methods used were 2.1% versus 24.3% and 0.1% versus 9.1% for sewage and river water samples, respectively. Sequence comparison studies indicated a high level of nucleotide identity in the G1, G2, G3, G4, G8 VP7, and P[4], P[6], and P[8] VP4 gene sequences between strains from the environment and those from patients in the region. This is the first environmental-based study on the G and P genotypes diversity of rotavirus in municipal wastewater and their receiving rivers in this geographical region. The high similarity between environmental and clinical rotavirus strains suggests both local circulation of the virus and potential exposure risks. In addition, it highlights the usefulness of sewage surveillance as an additional tool for an epidemiological investigation, especially in populations that include individuals with subclinical or asymptomatic infections that are precluded in case-based studies.

**Supplementary Information:**

The online version contains supplementary material available at 10.1007/s12560-024-09598-z.

## Introduction

Rotaviruses, known for their high genetic and antigenic diversity, remain a significant etiology of severe acute non-bacterial gastroenteritis in under-five-year children worldwide (WHO, 2021). Rotavirus accounts for approximately 258 million cases of childhood diarrhea with death cases estimated at 215,000 annually (Troeger et al., 2018). These naked, 11-segmented double-stranded RNA viruses have 10 distinct, fully differentiated genetic groups (A to J, L) based on the antigen specificities of the intermediate capsid protein (Omatola & Olaniran, 2022a). The group A rotaviruses are the commonest of the groups (A–C) causing diarrhea in humans and have been characterized as G and P genotypes based on the nucleotide sequence differences of genes 9 and 4 encoding the VP7 and VP4, respectively. To date, not less than 36 G and 51 P genotypes have been typed by the methods of reverse transcription-PCR (RCWG, 2021), and six major circulating G/P genotype combinations (G12P[8], G9P[8], G4P[8], G3P[8], G2P[4], and G1P[8]) are linked with rotavirus disease burden worldwide (Seheri et al., 2018).

The seasonal pattern of rotavirus infections differs from one geographical region to the other. In the tropics, rotavirus infects children all year round, though with fluctuations characterized by peaks and valleys. On the contrary, the incidence of rotavirus infection in the temperate region is almost zero in certain months, but peaks during the fall and winter (Lestari et al., 2020; WHO, 2021). In consideration of many animal host species susceptible to rotavirus infections that may serve as reservoirs for human rotavirus infection, increased human–animal cohabitation behavior, and the multiple exposure pathways involving fecal–oral route via person-to-person contacts or ingestion of fecally contaminated food and water, the complete eradication of the virus can be challenging (Omatola & Olaniran, 2022b, 2022c). In the aquatic environment, rotavirus adsorbs to particulate matter, in which state, both survival and persistence are enhanced (Naqvi et al., 2020). Thus, it is not surprising that the occurrence of rotavirus is frequently reported in association with wastewater, groundwater, freshwater sources, filter feeders (e.g., oysters and mussels), and vegetables garnered from contaminated water (Naqvi et al., 2020; Omatola & Olaniran, 2022b). Sewage remains an important environmental reservoir and environmentally mediated gateway for rotavirus infection. Human contact with rotaviruses in sewage or contaminated water, even at a low dose, can lead to infection because of the high degree of contagion which has been demonstrated in a dose–response model (da Silva et al., 2017). In the aquatic environment, rotavirus may persist at high levels regardless of the use of decontamination processes currently available for drinking water and sewage treatment (Kotwal & Cannon, 2014). Rotavirus, despite their inability to multiply outside the living host, can persist in the environmental reservoirs for extended periods, longer than most intestinal bacteria, thus, increasing the period during which they can be transmissible (Omatola & Olaniran, 2022b; WHO, 2018). Importantly, viral stability in the aquatic environment implies an increasing need for routine monitoring in aquatic hotspots.

In South Africa, rotavirus-associated morbidity remains a disease of public health concern as most children are infected before their third birthday (Asowata et al., 2018). Studies in the post-vaccination era have identified rotavirus as a major cause of childhood diarrheal and hospitalization in the country despite the early implementation and continuous use of the rotavirus vaccine (Asowata et al., 2018; Rossouw et al., 2021). Nearly four decades of meta-analysis findings documented an increased number of rotavirus cases in some provinces including KwaZulu-Natal (21% to 28%) and Western Cape (18% to 24%) post-vaccination (Omatola et al., 2021). In a recent gastrointestinal outbreak investigation in Northern Cape and KwaZulu-Natal province, rotavirus was identified in over 50% of the diarrheal cases. The outbreak was characterized by heterotrophic genotype G3P[8], G9P[8], and G2P[6] predominance while the vaccine G1P[8] genotype was least detected. Although the environmental source of the two outbreaks could not be ascertained, reasons such as delayed vaccination, suboptimal rotavirus vaccine coverage, and reduced vaccine efficacy were suggested in association with the rotavirus predominance in diarrhea-associated hospitalizations (Shonhiwa et al., 2020). Furthermore, rotavirus contamination through sewage-polluted water had been described in association with waterborne gastroenteritis, which negatively impacted the vulnerable population (Sekwadi et al., 2018).

The molecular profiles of rotavirus in wastewater and/or river water have been widely explored for genotype prediction and to correlate with circulating clinical strains in many countries including Spain (Silva-Sales et al., 2020), Italy (Ruggeri et al., 2015), China (Zhou et al., 2016), and Thailand (Kittigul & Pombubpa, 2021). In the study area, there is a dearth of information on rotavirus strains circulating in urban wastewater. The more recent study by Asowata et al. (2018) in the region, though reported a high rate of rotavirus infection among under-five children and identified the circulating strains, was case based, and circulating strains are more likely underestimated since it was dependent on subjects' age and disease severity. In the current study, we hypothesized that a knowledge gap exists between the rotavirus strains circulating in humans and the environmental reservoirs in the Durban area. Wastewater-based epidemiology remains a powerful tool to identify potential threatening strains (Ruggeri et al., 2015), predict possible emergence/reemergence of uncommon or unusual circulating rotavirus genotypes (Silva-Sales et al., 2020), and understand the actual rotavirus disease incidence in a given population since the virus particles can be excreted in feces of infected persons regardless of their symptoms. Usually, the final effluents from sewage treatment plants are discharged into the aquatic milieus servicing the city of Durban. Although the effluent discharges into rivers are generally believed to be of good quality, reports of underperformance of the treatment plants due to excessive stormwater loads, occasional shortage of chlorine, and aging infrastructure have been documented (Kretzmann et al., 2021). The frequent discharge of raw and poorly treated sewage effluents into most Durban rivers may pose a direct threat to the provision of microbiologically safe water. With the increase in population dynamics and the associated increase in dependence on diminishing water resources, an increase in the quantity of municipal wastewater produced on a daily basis is likely. Thus, current wastewater-based surveillance (WBS) for circulating rotavirus G and P types is warranted to generate information that could be used to inform vaccine efficacy and compare with published data on the types associated with clinical infections from the region. Further, the genotype data will highlight the possibility of transmission to the human population and provide an early warning system for incipient outbreaks of enterically transmitted rotaviruses from both symptomatic and non-symptomatic individuals in the Durban area. Additionally, we sequenced representative rotavirus VP7 and VP4 genes from sewage and associated rivers to establish the nucleotide sequence, compare with reference strains from the Genbank, and further perform the phylogenetic analysis to identify genetic clusters/lineages among different rotavirus strains.

## Materials and Methods

### Sample Collection and Processing for Viral Recovery

Two liters of raw, activated sludge and final effluent samples were collected at four municipal wastewater treatment plants (WWTP) under eThekwini Metropolitan Municipality in the city of Durban, situated in the Kwazulu-Natal province, South Africa. The WWTPs processes entail primary sedimentation, biological treatment (activated sludge or biofilter-based technology), and chlorination. The sampling areas were Northern Wastewater Treatment Plant (NWWTP) (29°48′49.356″S 30°52′23.7″E), Umbilo Wastewater Treatment Plant (UWWTP) (29°50′45″S 30°53′27″E), Phoenix Wastewater Treatment Plant (PWWTP) (33°25′29″N 112°6′40″E), and Isipingo Wastewater Treatment Plant (IWWTP) (29°59′24.936″S 30°54′21.78″E). The sampling position sites were determined using the global positioning system (GPS). The NWWTP, UWWTP, PWWTP, and IWWTP treat approximately 60–70, 23, 25–50, and 10.98–20 million liters per day of raw sewage, respectively, from the inhabitants of Newlands East, Pinetown, Phoenix, and Umlazi (https://www.boschholdings.co.za/project/northern-and-phoenix-wastewater-treatment/). Three of the plants (NWWTP, UWWTP, and PWWTP) operate via the conventional activated sludge treatment modality while the IWWTP employs biofilter for wastewater treatment. The IWWTP, NWWTP, UWWTP, and PWWTP discharge their treated effluent into the Isipingo, uMgeni, Umbilo, and Phoenix rivers, respectively (Bakare & Adeyinka, 2022). In addition to the samples from WWTPs, 2 L of river water was also collected at two different points (upstream and downstream) of the sewage-receiving rivers (Phoenix, Isipingo, Umbilo, and Umgeni rivers). These rivers are used for recreational activities, irrigation of agricultural crops, fish farming, and in some cases, sources of raw water for drinking water production by the inhabitants in the area. At all the locations, sampling was repeated at about four-week intervals between August and October 2021. The sewage and river water samples were briefly centrifuged for 30 min at 3000×*g* and 4 °C to remove suspended material and then subjected to skimmed-milk flocculation concentration procedures previously described elsewhere (Calgua et al., 2008). Briefly, a pre-flocculated skimmed-milk solution made by dissolving 10 g of skimmed milk powder in 1 L of autoclaved distilled water was pH adjusted with 1N HCL to 3.5. Twenty milliliters of the resulting solution were added to each of the 2 L water samples, which were also pH adjusted to 3.5. Samples were then stirred (8 h, room temperature) followed by flocs precipitation through gravity for another 8 h. Supernatants were removed such that the sediments were not dislodged, and the final volume was centrifuged (7000×*g*, 30 min, 4 °C). The pellets were resuspended in 2 mL of 1× PBS, adjusted with 1 N NaOH to pH 7.4, and immediately processed for viral nucleic acid extraction or stored at − 80 °C until assayed. Thus, to assess the efficiency of rotavirus recovery from the skimmed-milk flocculation concentration procedures, 2 L of deionized water was spiked with 1 mL of commercial Simian rotavirus (SA-11) stock suspension, and one unseeded sample was used as a procedural negative control. Both the seeded and unseeded samples were run in triplicates using the quantitative real-time PCR (RT-qPCR) after the concentration procedure described above. Rotavirus recovery rate (%) was determined by comparing the proportion of virus in the concentrate to the viral loads of those after the concentration procedure (Omatola et al., 2022).

### RNA Extraction, cDNA Synthesis, and RT-PCR of NSP3-Coding Gene Fragment of Rotavirus

In every 1 mL of viral concentrates, two different parallel RNA extractions were performed for each sample using the total RNA isolation kit (Thermo Fisher Scientific Inc., Waltham, Massachusetts, USA; Catalog No. #K0731) as per the manufacturer’s purification procedures and the total RNA from 2 extractions was resuspended in 50 µL of RNase-DNase-free H_2_O. Five microliters of RNA aliquot extracted from the sewage and river water samples were incubated (95 °C for 5 min) to denature the dsRNA genome and then chilled on ice for 3 min (Gouvea et al., 1990; WHO, 2009). The complementary DNA (cDNA) was synthesized from the resulting RNA solution using the RevertAid cDNA synthesis kit (Thermo Fisher Scientific Inc., Waltham, Massachusetts, USA; Catalog No. #K1632) according to the manufacturer's instructions. The primers reported by Pang et al., (2004) were used for reverse transcription-PCR to amplify the 87-bp NSP3 fragment of group A rotavirus (Table 1) (Supplementary Fig. 1). The 20 µL total reaction volume included 2.5 µL of template cDNA, 1 µL of 10 µM each of NSP3-F and NSP3-R primers, 1.2 µL of 25 mM MgCl_2_, 0.4 µL of 10 mM deoxynucleoside triphosphates mix, 2 μL of 10× PCR buffer, 0.1 μL of *Taq* DNA polymerase (Thermo Fisher Scientific Inc., Waltham, Massachusetts, USA), and 11.4 nuclease-free water. The cycling conditions were as indicated in Table 1, with the addition of an initial 3-min denaturation step at 94 °C and a final extension step at 72 °C for 5 min.

**Table 1:** Rotavirus NSP3 and the G and P typing PCR primers and conditions

Targets	Primer	Sequence (5′–3′)	Amplicon (bp)	References	Reaction conditions							
					Denaturation			Annealing		Extension		No cycles
					*T* (°C)	Time (s)		*T* (°C)	Time (s)	*T* (°C)	Time (s)	
NSP3	NSP3-F	ACCATCTACACATGACCCTC	87	Pang et al., 2004	94		30	60	30	72	60	40
	NSP3-R	GGTCACATAACGCCCC										
VP7 (G-typing)												
1st round												
	VP7-F	ATGTATGGTATTGAATATACCAC	881	Iturriza-Gomara et al., 2004	94		50	48	60	72	60	35
	VP7-R	AACTTGCCACCATTTTTTCC										
2nd round												
	aBT1 (G1)	CAAGTACTCAAATCAATGATGG	618	Gouvea et al., 1990	94		60	44	60	72	60	30
	aCT2 (G2)	CAATGATATTAACACATTTTCTGTG	521	Gouvea et al., 1990	94		60	44	60	72	60	30
	aET3 (G3)	CGTTTGAAGAAGTTGCAACAG	682	Gouvea et al., 1990	94		60	44	60	72	60	30
	aDT4 (G4)	CGTTTCTGGTGAGGAGTTG	452	Gouvea et al., 1990	94		60	44	60	72	60	30
	aFT9 (G9)	CTAGATGTAACTACAACTAC	179	Gouvea et al., 1990	94		60	47	30	72	60	30
	G12	CCGATGGACGTAACGTTGTA	266	Iturriza-Gomara et al., 2000	94		60	44	60	72	60	30
	VP7-R	As above										
VP4 (P-typing)												
1st round												
	con 2	ATTTCGGACCATTTATAACC	876	Gentsch et al., 1992	94		45	50	60	72	60	35
	con 3	TGGCTTCGCTCATTTATAGACA										
2nd round												
	2 T-1	CTATTGTTAGAGGTTAGAGTC	483	Gentsch et al., 1992	Same as first round							
	3 T-1	TGTTGATTAGTTGGATTCAA	267	Gentsch et al., 1992								
	1 T-1	TCTACTTGGATAACGTGC	345	Gentsch et al., 1992								
	con 3	As above										

*T* temperature, *sec* seconds, *bp* base pairs

### Semi-nested Multiplex RT-PCR for G Genotyping

All the 65 samples that were positive on conventional RT-PCR 87-bp assay were analyzed for VP7 (G-type) and VP4 (P-type) based on genotyping procedures previously described by Iturriza-Gomara et al. (2004) and Gentsch et al. (1992), respectively. The G genotyping of rotavirus was carried out using multiplex nested RT-PCR previously described by Iturriza-Gomara et al. (2004) but with modifications. In the first amplification reaction, genome segment 9 (defining VP7) was amplified with VP7-F and VP7-R generic primer pairs (Table 1). In a total final 25 μL volume, 2.5 μL of viral cDNA was added to a PCR mix containing 1.88 μL of 25 mM MgCl_2_, 0.5 μL of 10 mM deoxynucleoside triphosphates mix, 2.5 μL of 10 × PCR buffer, 0.13 μL of Taq DNA polymerase, 1.25 μL each of the forward VP7-F (10 µM) and the reverse VP7-R (10 µM) primers, and 14.99 μL of nuclease-free water. In the nested round of amplification, the PCR was multiplexed using the reverse VP7-R primer and the VP7-F (G-type) specific primer sequences (Table 1). The final PCR mixture comprised 1 µL of 50-fold diluted first-round PCR product showing visible bands or 5 µL template in the absence of visible bands, 6.5  µL of primer cocktail (i.e. 0.2 µM each of VP7-R and gene-specific primers), 4 µL of 25 mM MgCl_2_, 1 µL of 10 mM deoxynucleoside triphosphates mix, 4.8 μL of 10× PCR buffer, and 0.2 μL of *Taq* DNA polymerase. Nuclease-free water was used to make up the 50 µL final volume. The cycling conditions were as indicated in Table 1, with the addition of an initial 3-min denaturation step at 94 °C and a final 7-min extension step at 72 °C for each round. Both the first- and second-round PCR products were analyzed in 1.2% agarose gel that was stained with ethidium bromide after UV-trans-illumination (Supplementary Fig. 1). A 100 bp DNA ladder (GeneRuler™; Thermo Fisher Scientific Inc., Waltham, Massachusetts, USA) was employed as a molecular weight standard.

### Semi-nested Monoplex RT-PCR for G9 Typing

Samples that tested negative for rotavirus G9 on agarose gel after the above multiplex reaction were re-analyzed using a semi-nested monoplex RT-PCR employing the G9 defining primer and the reverse (VP7-R) primer with a predicted amplicon size of 179. In a final 25 reaction volume, 1 μL (50-fold diluted) first-round PCR product was included with 0.5 µL of 10 µM each of aFT9 and VP7-R primers, 1.5 µL of 25 mM MgCl_2_, 0.5 µL of 10 mM dNTP mix, 2.5 μL of 10 × PCR buffer, 0.13 μL of Taq DNA polymerase, and 18 nuclease-free water. The thermal cycling profiles were the same as for the other G-types except for the annealing conditions (Table 1).

### Semi-nested multiplex RT-PCR for P genotyping

Rotavirus P-typing was performed as previously described (Gentsch et al., 1992) using the VP4-consensus primers—con-2 and con-3 for the first-round PCR (Table 1). The final 20 μL reaction volume consisted of 4 μL viral cDNA, 1.5 μL of 25 mM MgCl_2_, 2 μL of 10× PCR buffer, 0.4 μL of dNTP mix, 0.5 μL of 10 µM each of con-2/con-3 primers, 0.2 μL of Taq DNA polymerase, and 9.9 μL DNase/RNase-free water. In the nested PCR step, 2.5 µL of the first-round PCR product was included in a PCR mix containing 1.5 µL of 25 mM MgCL2, 2.5 µL of 10× PCR buffer, 0.5 µL of dNTP mix, 0.63 µL of 10 µM each of VP4-specific forward primers mix, 0.63 µL of 10 µM con-3 reverse primer, 0.13 µL of Taq DNA polymerase, and DNase/RNase-free water to make 25 µL total volume. For both the first and second rounds of PCR, the cycling profiles were the same as indicated in Table 1, with the addition of an initial 3-min denaturation step at 94 °C and a final 7-min extension step at 72 °C for each round.

### Sequencing and Phylogenetic Analysis

Seventy randomly selected RT-PCR products were characterized by nucleotide sequencing in both directions with PCR oligonucleotide primers (G amplicons: VP7-F and VP7-R; P amplicons: con-3 and con-2) on an automated dideoxynucleotide method (Sanger sequencing) at Inqaba Biotechnical Industries (Pretoria, South Africa). BioEdit 7.1.3.0 was used for the analysis of each chromatogram and alignment of the sequence. A BLAST (Basic Local Alignment Search Tool) was employed as described (www.ncbi.nlm.gov/BLAST) to compare the nucleotide sequences with available sequences in the NCBI database. All relevant nucleotide sequences obtained from the BLAST were included in comparisons to gain insight into the extent of variability in rotavirus strains circulating in the area. Several sequences from those with the highest nucleotide sequence identity by BLAST search were included in the construction of phylogenetic trees. Phylogenetic trees were constructed with the rooted neighbor-joining algorithm, using the Kimura 2-parameter (K2-P) model with MEGA11 (Molecular Evolutionary Genetics Analysis) software. Bootstrapping test with 1000 replicates was used to assess the statistical reliability of the phylogenetic trees.

### Nucleotide Sequence Accession Numbers

Rotavirus nucleotide sequences obtained in the present study have been submitted to GenBank with access numbers OQ291159 to OQ291171 and OQ383132 to OQ383182.

## Results

**Fig. 1:**
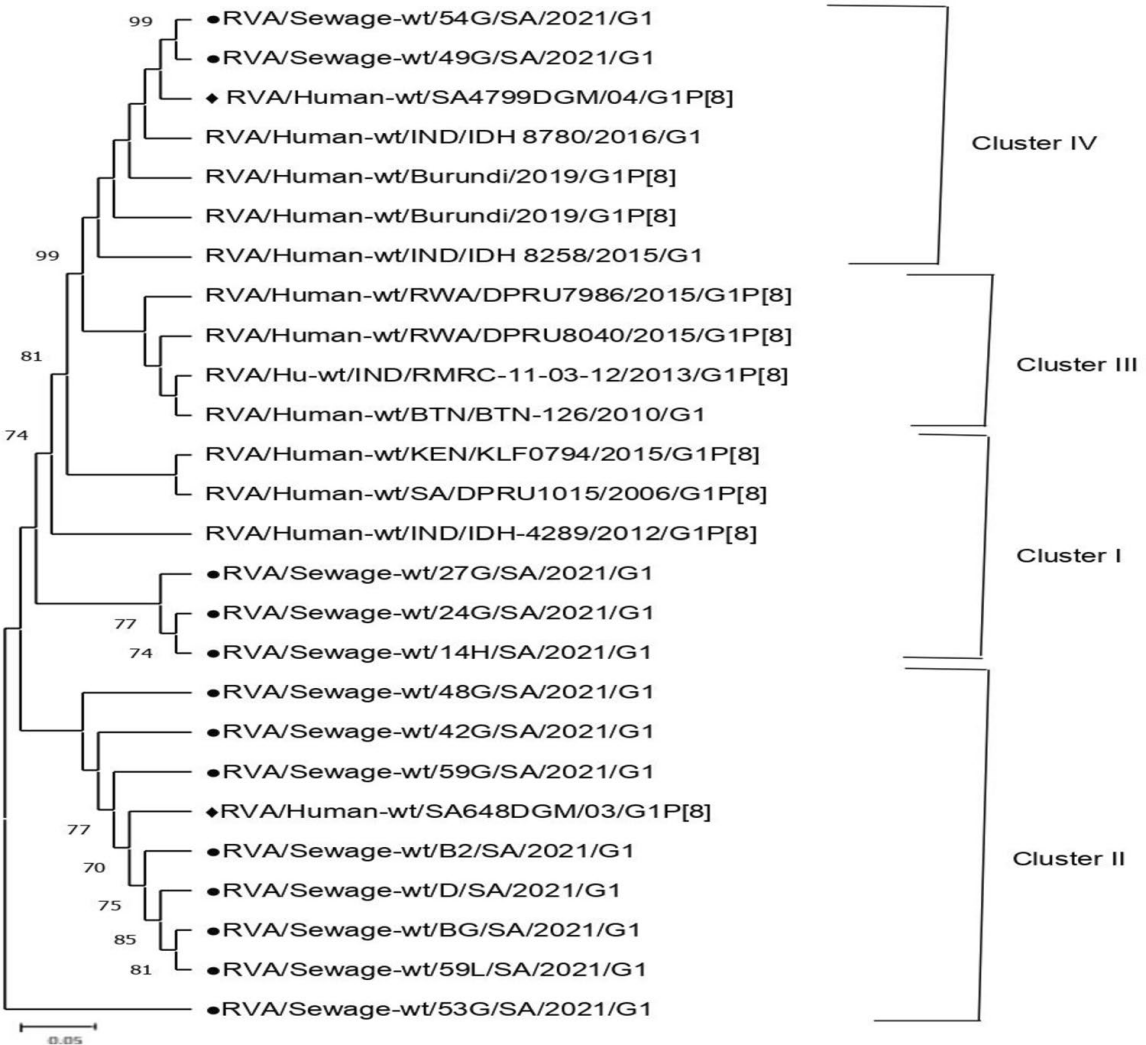
Phylogenetic relationship of VP7 nucleotide sequences of genotypes G1. Length of each G1 nucleotide sequence: 525 bp. Assignment into clusters was as described by Intamaso et al. (2017) and Zhou et al. (2016). All nucleotide sequences obtained from GenBank are named as described previously by Matthijnssens et al. (2008). Sequences derived from sewage and reference clinical strains from South Africa were marked by filled circles (●) and filled diamonds (♦), respectively. The scale bar beneath the phylogenetic tree represents the number of nucleotide substitutions or variations/sites. The percent bootstrapped support (1000 replicates) is designated by numbers at the branch nodes. In each case, bootstrapped values less than 70 were not shown

**Fig. 2:**
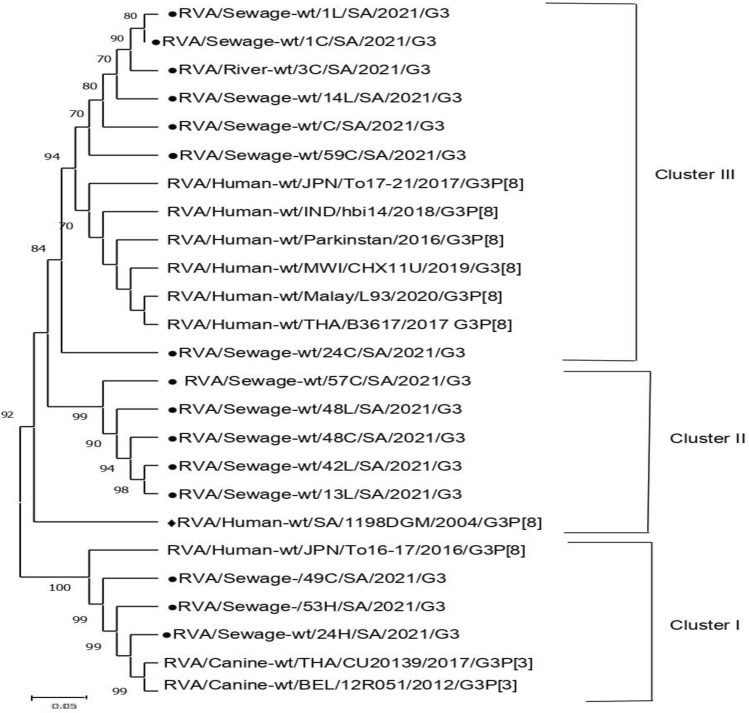
Phylogenetic relationship of VP7 nucleotide sequences of genotypes G3. Length of each G3 nucleotide sequence: 840 bp. Assignment into clusters was described by Intamaso et al. (2017) and Zhou et al. (2016). All nucleotide sequences obtained from GenBank are named as described previously by Matthijnssens et al. (2008). Sequences derived from sewage and reference clinical strains from South Africa were marked by filled circles (●) and filled diamonds (♦), respectively. The scale bar beneath the phylogenetic tree represents the number of nucleotide substitutions or variations/sites. The percent bootstrapped support (1000 replicates) is designated by numbers at the branch nodes. In each case, bootstrapped values less than 70 were not shown

**Fig. 3:**
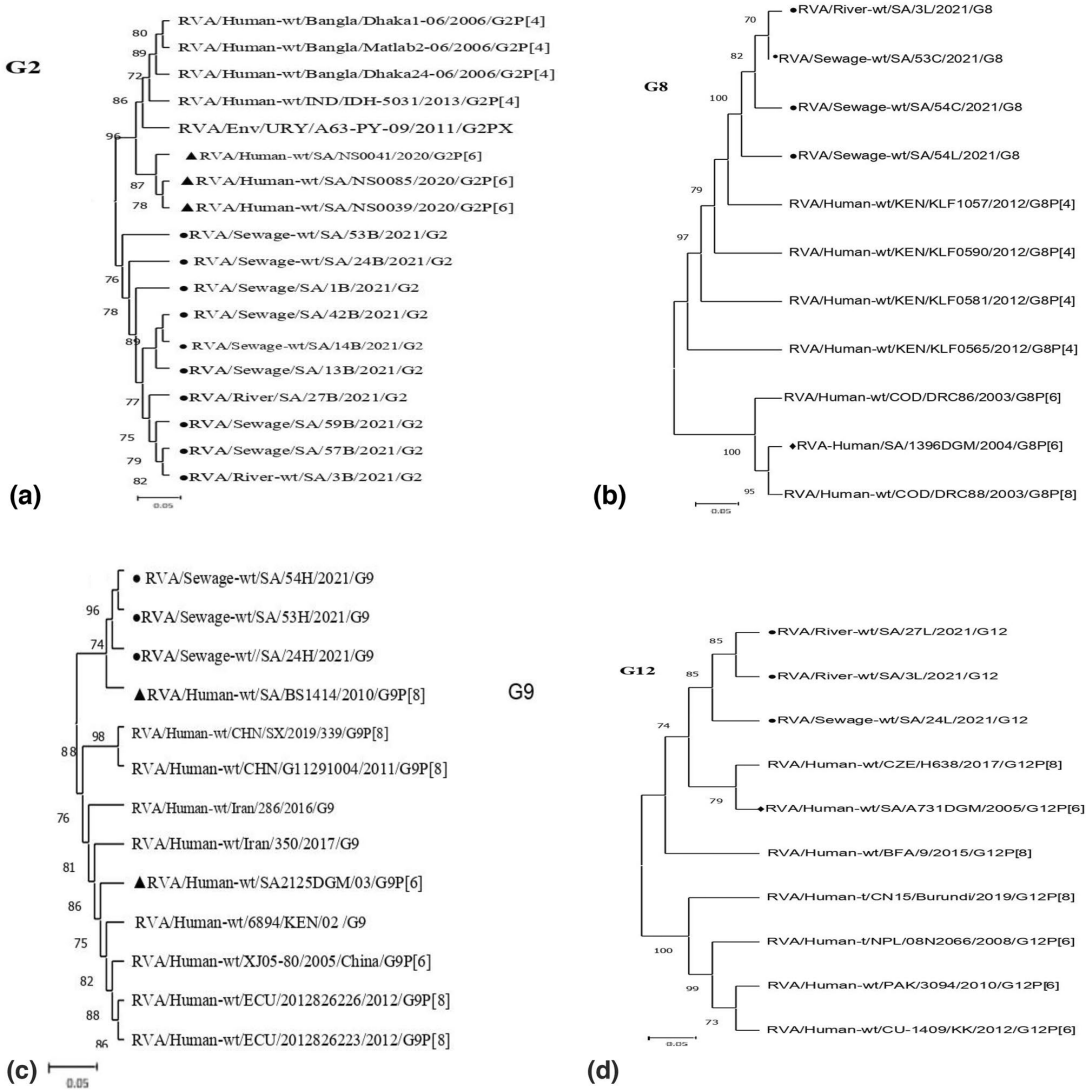
Phylogenetic relationship of VP7 nucleotide sequences of genotypes G2 (**a**), G8 (**b**), G9 (**c**), and G12 (**d**). Nucleotide sequence lengths: Each G2 = 747 bp, G8 = 831 bp, G9 = 516 bp, and G12 = 565 bp. All nucleotide sequences obtained from GenBank are named as described previously by Matthijnssens et al. (2008). Sequences derived from sewage and reference clinical strains from South Africa were marked by filled circles (●) and filled diamonds (♦), respectively. The scale bar beneath the phylogenetic tree represents the number of nucleotide substitutions or variations/sites. The percent bootstrapped support (1000 replicates) is designated by numbers at the branch nodes. In each case, bootstrapped values less than 70 were not shown

**Fig. 4:**
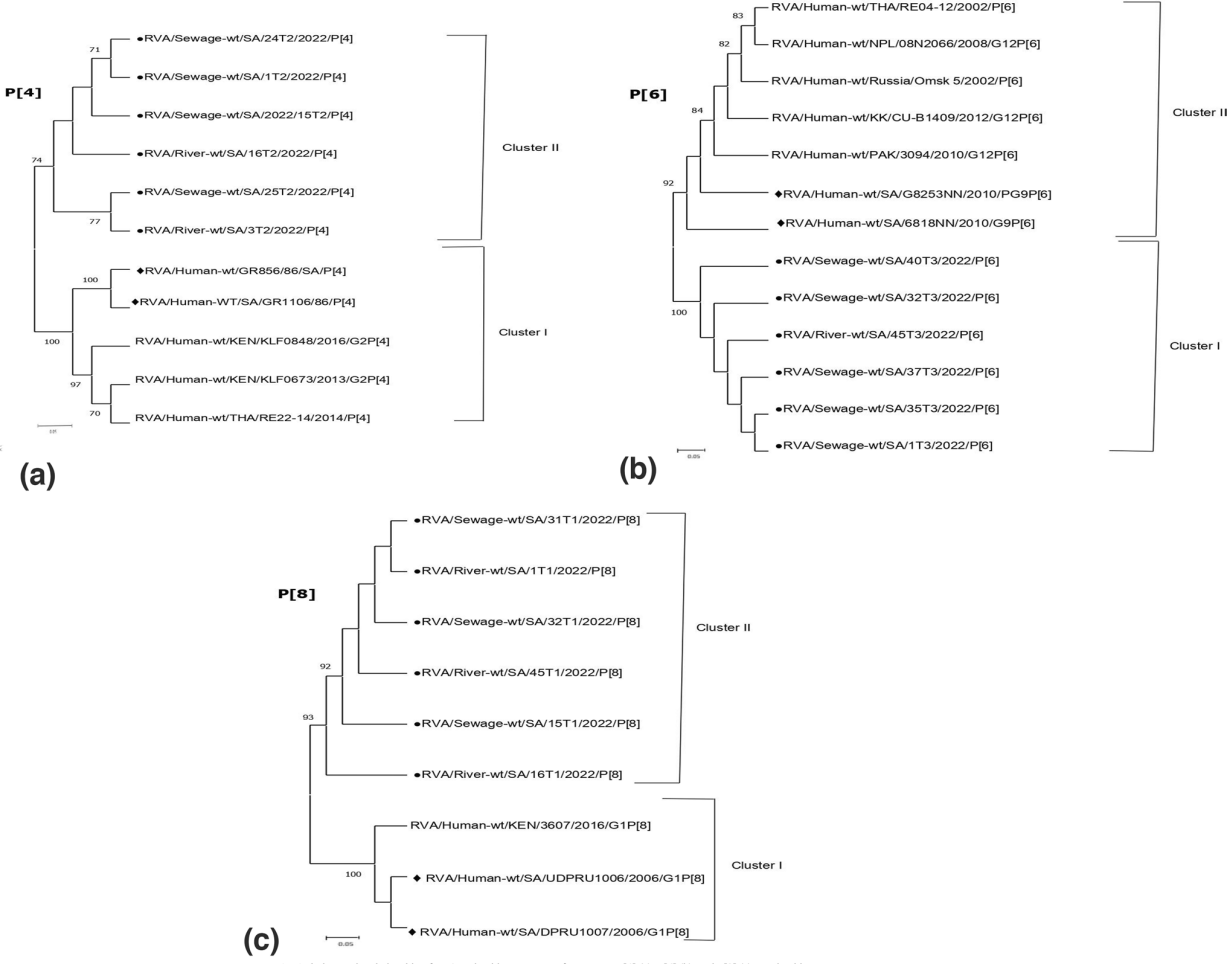
Phylogenetic relationship of VP4 nucleotide sequences of genotypes P[4] (**a**), P[6] (**b**), and P[8] (**c**). Nucleotide sequence lengths: Each P[4] = 816 bp, P[6] = 828 bp, and P[8] = 839 bp. All nucleotide sequences obtained from GenBank are named as described previously by Matthijnssens et al. (2008). Sequences derived from sewage and reference clinical strains from South Africa were marked by filled circles (●) and filled diamonds (♦), respectively. The scale bar beneath the phylogenetic tree represents the number of nucleotide substitutions or variations/sites. The percent bootstrapped support (1000 replicates) is designated by numbers at the branch nodes. In each case, bootstrapped values less than 70 were not shown

In the current study, 65 out of the total 69 samples tested positive for rotavirus. Sixty-four (VP7) G genotypes (49 in sewage and 15 in rivers) and 63 (VP4) P genotypes (41 in sewage and 22 in rivers) were characterized, belonging to 7 G- (G1, G2, G3, G4, G8, G9, and G12) and 3 P genotypes (P[3], P[4], and P[8]), respectively (Tables 2, 3, 4 and 5). Genotype G1 accounting for 26.6% (17/64) predominates in both the sewage (24.5%, 12/49) and river (33.3%, 5/15) samples. In the sewage samples, the second most prevalent G genotype was G3 (22.4%, 11/49), followed by G2 (14.3%, 7/49), G4 (12.2%, 6/49), G12 (10.2%, 5/49), G9 (8.2%, 4/49), and G8 (6.1%, 3/49). Mixed G1 + G3 was detected in 2.0% of the samples on nested RT-PCR genotyping (Table 2) (Supplementary Fig. 1). In the river water samples, genotypes G2 and G4 with equal detection rates (20.0%, 3/15) were the second most common genotype, followed by G3 and G12 with percentages of 13.3% each, while G8 and G9 were not detected (Table 4). In general, the relative distribution of VP4 (P-type) strains identified in the sewage samples was consistent with the genotype trends in river samples. Genotype P[4] accounting for overall VP4 prevalence at 39.7% (25/63) was the most common genotype circulating in both the sewage (36.6%, 15/41) and river water (45.5%, 10/22) samples. Genotype P, [6] which accounts for an overall VP4 prevalence of 30.2% (19/63), was the second most frequent genotype with corresponding rates at 29.3% (12/41) and 31.8% (7/22) in sewage and river samples, respectively. Similarly, genotype P[8] with a total P prevalence of 11.1% (7/63) was the least-detected genotype in both sewage and river samples. Mixed genotypes (P[6] + P[8]) accounted for 24.4% (10/41) and 9.1% (2/22) of the strains in sewage and river samples, respectively (Tables 3 and 5). In addition, the nucleotide sequences from 65 samples in the study were compared with South African and other reference strains reported globally (Table 6), to understand the extent of variability in rotavirus strains circulating in the Durban area. The phylogenetic relationships of VP7 (G) strains are depicted in Figs. 1, 2, 3 and 4. All the rotavirus G1 strains sequenced showed a high degree of nucleotide sequence similarity in the VP7 gene (97.5–99.8%) and were found in three clusters (Table 6). The two G1 strains in cluster IV shared an extremely high degree of nucleotide sequence identity (99.8%) between them (Supplementary Fig. 2) and with reference strains located in the same cluster which were previously identified in India (MK829372, 99.1–99.5% identity), Burundi (MK685890, MK685895, 98.8–99.5% identity), and South Africa (GQ338879, 98.6–98.8% identity). The G1 strains in clusters I and II showed more than 98% and 96% sequence identity, respectively, to each other. In the latter cluster, the strains were co-clustered with a previously detected human G1 strain among South African children (GQ338876) and were closely related (94.9–95.1% identity) (Fig. 1). The G3 VP7 gene sequences exhibit a high level of sequence divergence (78–100%) among themselves and can be divided into three clusters (I–III) phylogenetically. The reference strains LC477363, MH560415, KX681826, ON791968, LC541502, and MN836863 and six of the environmental G3 strains were genetically highly related (98.2–98.8% nucleotide sequence identity) and were clustered in the same lineage phylogenetically (Fig. 2). Also, a reference human strain from South Africa (GQ338880) exhibited a high nucleotide sequence relationship with G3 strains in cluster II (98.2%) and great sequence divergence from the equine strains in cluster I (96–98% vs. 77–79%). Three of the G3 sequences from sewage in cluster I displayed more nucleotide sequence identity with reference strains from a canine (KF614063 and MT364862) than classical human G3 strain from Japan (LC542015) (96–98% vs. 85–86%) on the same main phylogenetic branch (Fig. 2). The G2 sequences, which clustered in a separate branch, displayed very high (96–99%) nucleotide sequence similarities with the globally spreading reference G2P [4] clinical strains detected in Bangladesh (EF690789/90/92, 98% identities) and Eastern India (KM581040, 97–99% identities). Furthermore, the G2 sewage strains were 96–98% genetically related to the human G2P[6] previously reported in South Africa (MW392050, MW392051, MW392053) (Fig. 3). A human G12 reference rotavirus strain from South Africa (GQ338891), Burkina Faso (MN758626), and the Czech Republic (MK690524) shared 95.8–97.4% genetic background identity with a G12 VP7 gene sequence from a Phoenix South African river. Similarly, a clinical G9P[8] strain was previously detected in South Africa (DQ822599), and all the G9 sewage strains were clustered within the G9-III lineage on a separate main phylogenetic branch. The uncommon G8 strains, which were identified through sequencing displayed 95.8–96% sequence identity with the human G8P[6] strain recently detected in Northern Pretoria, South Africa (GQ344620) (Fig. 3). Figure 4 shows the phylogenetic relationships of rotavirus VP4 (P) gene sequences. All P[4] strains sequenced exhibited high sequence identity (97.1–99.8%) to each other irrespective of their sample source and were more closely related to two reference clinical strains detected in Kenya (MZ096103, MZ094854, 98.1–98.7% identity) and a Thailand strain (MW075445, 98.8–99.3% identity). In contrast, the P[4] sequences from both sewage and rivers displayed lower identities (84.5–85%) in the VP4 gene to two human P[4] strains from South Africa (AF170840, AF170841). The P[6] rotaviruses from different sewage sources and rivers were extremely related to each other (99.6–100%) in the VP4 gene sequence and are co-clustered phylogenetically. Additionally, these sequences of both sewage and river origin had 95.9–100% sequence similarity with the human P[6] reference strain recently detected in South Africa (AF529875/6), Pakistan (KY497521), Russia (GU226767), Nepal (IC372903), and Thailand (MW075442) (Table 6). All the P[8] strains, regardless of the sample matrix, showed extremely high nucleotide sequence identity (99.0–100%) to each other and to the globally spreading human P[8] reference strains detected in South Africa (MT854358, MT854369, 92.0–93.3% identity) and Kenya (MH291322, 93.7–94.7% identity).

**Table 2:** Distribution of rotavirus VP7 (G) genotype in raw or partially treated sewage and final effluents of four Durban wastewater treatment plants

Genotype	No. (%) of positive samples								
	PWWTP		NWWTP		UWWTP		IWWTP		Total
	Raw/PT	Final effluent	Raw/PT	Final effluent	Raw/PT	Final effluent	Raw/PT	Final effluent	
G1	2 (22.2)	2 (33.3)	3 (30.0)	1 (11.1)	1 (33.3)	0 (0.0)	2 (25.0)	1 (25.0)	12 (24.5)
G2	1 (11.1)	1 (16.7)	2 (20.0)	1 (11.1)	1 (33.3)	0 (0.0)	0 (0.0)	1 (25.0)	7 (14.3)
G3	3 (33.3)	1 (16.7)	2 (20.0)	2 (22.2)	1 (33.3)	0 (0.0)	1 (12.5)	1 (25.0)	11 (22.4)
G4	1 (11.1)	0 (0.0)	1 (10.0)	1 (11.1)	0 (0.0)	0 (0.0)	3 (37.5)	0 (0.0)	6 (12.2)
G8	0 (0.0)	0 (0.0)	0 (0.0)	3 (33.3)	0 (0.0)	0 (0.0)	0 (0.0)	0 (0.0)	3 (6.1)
G9	0 (0.0)	1 (16.7)	1 (10.0)	1 (11.1)	0 (0.0)	0 (0.0)	1 (12.5)	0 (0.0)	4 (8.2)
G12	1 (11.1)	1 (16.6)	1 (10.0)	0 (0.0)	0 (0.0)	0 (0.0)	1 (12.5)	1 (25.0)	5 (10.2)
G1 + G3	1 (11.1)	0 (0.0)	0 (0.0)	0 (0.0)	0 (0.0)	0 (0.0)	0 (0.0)	0 (0.0)	1 (2.0)
Total	9	6	10	9	3	0	8	4	49

*NWWTP* Northern Wastewater Treatment Plant, *PWWTP*  Phoenix Wastewater Treatment Plant, *UWWTP* Umbilo Wastewater Treatment Plant, *IWWTP* Isipingo Wastewater Treatment Plant, *PT*  partially treated

**Table 3:** Distribution of rotavirus VP4 (P) genotype in raw or partially treated sewage and final effluents of four Durban wastewater treatment plants

Genotype	No. (%) of positive samples								
	PWWTP		NWWTP		UWWTP		IWWTP		Total
	Raw/PT	Final effluent	Raw/PT	Final effluent	Raw/PT	Final effluent	Raw/PT	Final effluent	
P[4]	3 (30.0)	2 (50.0)	2 (33.3)	1 (33.3)	4 (44.4)	2 (50.0)	1 (25.0)	0 (0.0)	15 (36.6)
P[6]	4 (40.0)	2 (50.0)	2 (33.3)	1 (33.3)	1 (11.1)	1 (25.0)	1 (25.0)	0 (0.0)	12 (29.3)
P[8]	1 (10.0)	0 (0.0)	1 (16.7)	0 (0.0)	1 (11.1)	0 (0.0)	1 (25.0)	0 (0.0)	4 (9.8)
P[6] + P[8]	2 (20.0)	0 (0.0)	1 (16.7)	1 (33.3)	3 (33.3)	1 (25.0)	1 (25.0)	1 (100)	10 (24.4)
Total	10	4	6	3	9	4	4	1	41

*NWWTP* Northern Wastewater Treatment Plant, *PWWTP*  Phoenix Wastewater Treatment Plant, *UWWTP* Umbilo Wastewater Treatment Plant, *IWWTP* Isipingo Wastewater Treatment Plant, *PT*  partially treated

**Table 4:** Frequency of rotavirus VP7 (G) genotype in Durban rivers receiving treated effluents from municipal wastewater treatment plants

Genotype	No. (%) of positive samples				
	Phoenix river	Umgeni river	Umbilo river	Isipingo river	Total
G1	2 (33.3)	2 (33.3)	1 (100)	0 (0.0)	5 (33.3)
G2	1 (16.7)	1 (16.7)	0 (0.0)	1 (50.0)	3 (20.0)
G3	1 (16.7)	1 (16.7)	0 (0.0)	1 (50.0)	2 (13.3)
G4	1 (16.7)	1 (16.7)	0 (0.0)	0 (0.0)	3 (20.0)
G9	0 (0.0)	0 (0.0)	0 (0.0)	0 (0.0)	0 (0.0)
G12	1 (16.7)	1 (16.7)	0 (0.0)	0 (0.0)	2 (13.3)
Total	6	6	1	2	15

**Table 5:** Frequency of rotavirus VP4 (P) genotype in Durban rivers receiving treated effluents from municipal wastewater treatment plants

Genotype	No. (%) of positive samples				
	Phoenix river	Umgeni river	Umbilo river	Isipingo river	Total
P[4]	3 (33.3)	3 (75.0)	4 (50.0)	0 (0.0)	10 (45.5)
P[6]	2 (22.2)	1 (25.5)	3 (37.5)	1 (100)	7 (31.8)
P[8]	2 (22.2)	0 (0.0)	1 (12.5)	0 (0.0)	3 (13.6)
P[6] + P[8]	2 (22.2)	0 (0.0)	0 (0.0)	0 (0.0)	2 (9.1)
Total	9	4	8	1	22

**Table 6:** A summary table with sequence identity percentages among study samples and between study sequences and prototypes

Sample strains		Percentage identity
	Among study samples	Reference strains
		Accession no (Country), Identity with samples
G1	97.5–99.8%	
Cluster I		MZ095676 (Kenya), MT854401 (South Africa), KM581023 (India), 98.5–98.9%
Cluster II		GQ338876 (South Africa), 94.9–95.1%
Cluster III		MN632920, MN633057 (Rwanda), KT387239, (India) AB905458, 95.4–99.2%
Cluster IV		MK829372 (India), MK685890, MK685895 (Burundi), GQ338879 (South Africa), 98.6–99.5%
G2	99.6–100%	EF690789/90/92 (Bangladesh), KM581040 (India), MW392050/51/53 (South Africa), 96–99%
G3	78–100%	
Cluster I		KF614063 (Belgium), MT364862 (Thailand), LC542015 (Japan), 84–98.3%
Cluster II		GQ338880 (South Africa), 98.2%
Cluster III		LC477363 (Japan), MH560415 (India), KX681826 (Pakistan), ON791968 (Malawi), LC541502 (Malaysia), and MN836863 (Thailand), 98.2–98.8%
G8	99.8–100%	GQ344620 (South Africa), 95.8–96%
G12	100%	GQ338891 (South Africa), MN758626 (Burkina Faso), MK690524 (Czech), 95.8–97.4%
P[4]	99.8–100%	MZ096103, MZ094854 (Kenya), MW075445 (Thailand), 98.1–99.3%, AF170840, AF170841 (South Africa), 84.5–85%
P[6]	99.6–100%	AF529875/6 (South Africa), KY497521 (Pakistan), GU226764 (Russia), LC372903(Nepal), MW075442 (Thailand), 95.9–100%
P[8]	99.0–100%	MT854358, MT854369 (South Africa), MH291322 (Kenya), 92.0–94.7%

## Discussion

The molecular detection and characterization of rotavirus in urban wastewaters have been largely explored across the globe to generate information on circulating genotypes, correlate the level of vaccine protection, and provide a signal of imminent outbreaks through the receiving water bodies in given populations. While most studies on rotaviruses in South Africa are case-based surveillance, few are environmentally based and none from the Durban area has provided evidence on environmental circulating genotypes and rotavirus phylogeography which are of great significance in environmental microbiology. Therefore, the epidemiological data in the current study represent the first documentation on G- and P- genotypes diversity of rotavirus concurrently in municipal sewage and sewage-receiving rivers in this geographical region. In South Africa, children under six years represent 14.4% of the total population (Galal, 2021), and considering that the brunt of hospital-associated pediatric diarrheal morbidity and deaths are due to rotavirus infection in these young ages, the group A rotaviruses detected in sewage and their receiving rivers were characterized through sequencing, genotyping, and phylogenetic analyses to reveal additional insight into the molecular profiles and epidemiologic relevance of virus strains circulating in the sewage-river water phases. Of a noteworthy observation, there was a high prevalence of clinically relevant rotavirus G and/or P genotypes in the effluents of the four treatment plants and corresponding genotype diversity in the sewage-receiving aquatic bodies.

In this study, sequence and phylogenetic analysis of common rotavirus VP7 G1, G2, and G3 strains showed high levels of nucleotide sequence similarities between environmental and clinical reference strains. These findings, depicting the close relationship between human and environmental rotavirus strains, indicate that environmental genomic surveillance could be a sensitive tool for tracking viral transmission pathways. Again, our findings highlight the useful application of environmental-based surveillance studies in areas where clinical-based surveillance is limited or at a low level. The predominance of G1 rotavirus in both sewage and sewage-receiving aquatic environments pinpoints to a high fecal shedding and continuous circulation of the strain in the Durban metropolis, allowing its detection regardless of the dilution in sewage and associated aquatic bodies. The high prevalence of circulating G1 virus in both sewage and river waters may pose a serious concern as most reported waterborne rotavirus outbreaks manifesting severe symptoms in people of all ages were caused by high G1P[8] genotype load in sewage-contaminated water sources (Ruggeri & Fiore, 2012). Although viral loss of viability and infectivity following chemical treatment procedures may reduce the potential risk associated with human or animal contacts as compared to the strains in humans. In comparison with our findings, Ruggeri et al. (2015) reported similar G1 predominance in sewage and concurrently among Italian children with acute rotavirus gastroenteritis. In a Philippian study where G1 rotavirus was identified as the second most frequent cause of childhood diarrhea, the infecting G1 strain was simultaneously recognized predominantly in river samples connected with municipal sewage (Imagawa et al., 2020). Sequence and phylogenetic analysis of the rotavirus VP7 gene delineated the majority of the G1 genotype from sewage sources into lineage II followed by lineage I and IV. The finding of the G1 virus in clusters I, II, and IV which contained a G1P[8] genotype combination previously reported in South Africa, Burundi, and India by Seheri et al. (2010), Cassien (2020), and Nayak et al. (2019), respectively suggested that the G1P[8] might be the predominant rotavirus G/P genotype combination currently circulating in the Durban area. This has been corroborated by a recent meta-analysis data of rotavirus genotype diversity in South Africa which indicated the predominance of G1P[8] genotype combination in 32% of rotavirus diarrheal cases (Omatola et al., 2021). Despite the overall G1 predominance that had been reported (Omatola et al., 2021) pre-and post-vaccine execution in South Africa, the epidemiologic shift from G1 to G2, G3, G9, or G12 in rotavirus cases had been observed in some South African provinces since vaccine licensure in August 2009 (Groome et al., 2014). The level of detected G2 and P[6] genotypes and the close genetic distance with the reference human G2P[6] genotype combination isolated at a close interval in the country imply that the genotype is prevalent in the area, in which case, the continuous circulation in the environmental reservoirs may be perpetrating chain transmission. The epidemiological relevance of rotavirus G2P[6] among diarrheic children in South Africa has been documented severally (Rossouw et al., 2021; Shonhiwa et al., 2020). Additionally, the G2 strains from sewage were found to exhibit extremely high genetic relatedness with clinical G2P[4] strain previously reported in Bangladesh (Mustafizur et al., 2007), and India (Nayak et al., 2019), an observation likely pointing to both local and global occurrence of the G2 strain detected in sewage.

Rotavirus G3 strains which account for the second and third most dominant strains detected respectively in sewage and rivers had three distinguishable evolutionary branches, which pinpoint to a tripartite G3 co-circulation chain in the local population. A study by Seheri et al. (2010) identified significant G3 strains in the Northern provinces, sequences of which displayed high phylogenetic relationships with G3 strains from our current sewage study. In the Durban area, Shonhiwa et al. (2020) in a diarrheal outbreak investigation documented the G3 predominance in rotavirus cases. In a more recent South African study involving pediatric hospital admitted cases, the G3 genotype in combination with P[8] was significantly recognized (Rossouw et al., 2021), observation likely suggesting the persistence of G3 strains which further supports the high G3 excretion confirmed in the current sewage surveillance study. The findings of extremely high sequence and phylogenetic relatedness of G3 strains in cluster I to animal strains previously described in Thailand (Charoenkul et al., 2021) and Belgium (Theuns et al., 2014) might be pointing to a similar ancestral origin when there is fecal contamination from animals. Rotavirus infection has a significant impact on both human and animal health (Omatola & Olaniran, 2022a). Considering the high zoonotic potential of rotaviruses, proper disposal of human and animal feces, as well as good water and environmental hygiene, may help reduce the potential risks of viral transmission via the fecal–oral route. Although the G3 strains from sewage did not display higher identities to the clinical as compared to animal strains, it is difficult for the current study to ascribe the G3 strains to animals or humans alone. This is because rotavirus infects more than one host, and several of the sequences could be found in the environmental samples. Notwithstanding, host-rotavirus strain specificity is well recognized globally. However, primary transmission and genome recombination events in a host could trigger unusual strains’ emergence. Hence, a case-based investigation in future studies is needed to show whether these were novel antigenic variants circulating in the Durban area. Again, the G3 strains from sewage and river in cluster III revealed high sequence and phylogenetic relatedness to clinical G3 strains recently identified in Japan (Fujii et al., 2020), Malawi (Mhango et al., 2022), Malaysia (Amit et al., 2021), and Thailand (Chansaenroj et al., 2020), indicating the likelihood of long-distance transmission among the strains which had been previously suggested (Du et al., 2021) for strains isolated at near intervals. In the study, our findings of G2 and G3 rotavirus occurring as dominant circulating strains in sewage and associated rivers, besides G1 strains, highlight the distribution of heterotrophic vaccine strains in the Durban area which have been corroborated by the recent findings of Omatola et al. (2021) in a nearly four decades meta-analysis studies among South African pediatric population with acute rotavirus gastroenteritis cases. The high frequency at which the heterotrophic strains were detected in the environmental sources, which may reflect endemic proportion in the human population, supports epidemiological surveillance data, indicating the emergence and the increased epidemiological fitness of these genotypes in clinical cases from other African countries (Seheri et al., 2018) and other countries in the world (Okitsua et al., 2022). The continuous excretion of these strains not fully covered in the Rotarix vaccine in national use might be explained by a lack of sufficient neutralization and capacity for intestinal replication, resulting in eventual shedding in feces. Although the Rotarix vaccine in national use has been shown to elicit both homotypic and heterotypic response (Omatola & Olaniran, 2022a), a reduction in protective vaccine-elicited immunity against the non-G1 strains circulating among diarrheic children in South Africa has been previously reported (Groome et al., 2014). Additionally, the high occurrence of G2 and G3 rotavirus in both sewage and water sources which strongly reflects the emerging types in clinical cases in the same geographical area during the licensure era (Shonhiwa et al., 2020), indicates that both sewage and water represent a potential reservoir and vehicle of viral infection.

Generally, rotavirus VP7 genes of less prevalent G8, G9, and G12 strains were phylogenetically closely related between environmental and clinical reference strains. The finding of these genotypes in sewage and their receiving rivers, albeit at low detection rates, suggests the circulation of the virus strain in the Durban area. The close phylogenetic relatedness of environmental strains (G8, G9, and G12) to the clinical strains identified in the country also suggests that they might have originated from the same ancestor, though not necessarily from the same pool of circulating genotypes. Although the less prevalent G9 and G12 strains have been previously reported to be among the major types associated with rotavirus diarrhea in the Durban area (Asowata et al., 2018; Shonhiwa et al., 2020), other regions in South Africa (Omatola et al., 2021), and worldwide (Okitsua et al., 2022), the low rate of genotypes detection in the present study could be attributed to temporal changes in circulating strains, low prevalence during the sampling period, or low virus titers due to dilution effects. Like in a Thailand study (Intamaso et al., 2017), the primers designed by Gouvea et al. (1990) displayed cross-reactivity between VP7, G12, and G8. Thus, DNA sequencing was needed for the confirmation of genotype G8 results. Rotavirus genotype G8 is typical of strains detected in bovine populations, though it has adapted rapidly to human populations since the first reports of human cases in 1980 (Matsuno et al., 1985). Since emergence, G8 rotavirus have been described in association with P[1], P[2], P[4], P[6], and P[8] globally (Omatola & Olaniran, 2022a). In the current study, all three unusual G8 strains which were isolated at a monthly interval from sewage sourced from the NWWTP facility were genetically and phylogenetically closely related to the human G8 strain in combination with the P[6] genotype reported in the area (Seheri et al., 2010), an observation probably suggesting a staged continuity of transmission in the locality during the period. In conformity with the low detection rate (6.1%) of the unusual G8 rotavirus genotype in the current sewage surveillance study, a 4% prevalence rate had been reported in the South African pediatric population in a meta-analysis study (Omatola et al., 2021) which attributed the zoonotic infection to possible inter-host transmission and genome segments reassortment events between human and bovine rotavirus. Although, the current water and sewage surveillance study may be an important epidemiologic tool to assess the circulation of unusual/rare rotavirus genotypes in the community, complementary analysis for rotaviruses of animal origin would be needed to uncover the full picture of actual rotavirus strains circulating in the environment.

In the current study, the finding of extremely high genetic similarities within VP4 P[4], P[6], and P[8] gene sequences from both sewage and rivers and their co-clustering on one main phylogenetic branch are suggestive of the circulation of single transmission lineage in these genotypes in the Durban area. The dominance of genotype P[4] in both sewage and their receiving rivers could be ascribed to the level of genotype prevalence in the human population, correlating equally well with our recent meta-analytic study, where the human genotype strain was found to have significantly evolved from 16% pre-vaccine era to 30% post-vaccine execution in South Africa. Again, the high rate of P[4] genotype observed in the current study is not unusual as the genotype emergence has been reported severally in licensure area, especially where Rotarix is used in the national pediatric immunization schedules (Carvalho-Costa et al., 2019; Omatola & Olaniran, 2022a). Additionally, the high genetic relatedness of P[4] genotypes detected in the study to the globally important human P[4] strains recently reported in a Kenyan (Lambisia et al., 2021) and Thailand (Kittigul & Pombubpa, 2021) study highlights the potential benefit of an environmental-based study as a complementary tool to clinical monitoring of rotavirus A infection or predict circulating genotypes in human samples during viral outbreaks.

This study has certain limitations. First, the multiplex RT-PCR typing system applied would only detect the most common rotavirus G and P types. However, several types would not be detected using the current methods and those types would, therefore, be missed. This limitation could be surmounted by using next-generation sequencing that can cover larger regions of the VP7 and VP4 genes. Second, the current study did not provide any indication of the infectivity of the viruses circulating in the environmental matrices, warranting its investigation by future studies. Of note, rotavirus in the environmental reservoirs may be exposed to different pressures and stresses which may lead to the viral loss of viability, infectivity, and transmissibility as compared to the strains in humans. Notwithstanding, our finding is important as human’s contact with, or ingestion of sewage-contaminated river water has been shown to constitute a potential health risk (Ruggeri & Fiore, 2012; WHO, 2020).

In the current study, rotavirus genotyping and phylogenetic study of VP7 genotype G1–G3, G8, G9, and G12 sequences uncovered high sequence identities between the environmental strains and previously identified clinical strains. Our findings suggest that rotaviruses in circulation in Durban municipal sewage and the receiving rivers closely mirrored the common strains frequently responsible for severe acute gastroenteritis in infants and young children, which support the involvement of common rotavirus genotypes in asymptomatic or subclinical infection of children and adults not necessitating hospital admission. Importantly, these findings suggest that molecular-based rotavirus surveillance of water and wastewater may be an important epidemiologic tool to assess the true community structure of rotaviruses from a population of different individuals regardless of their symptoms.

## Supplementary Information

Below is the link to the electronic supplementary material.

Supplementary file1 (DOCX 992 KB)

Supplementary file2 (DOCX 126 KB)

## Data Availability

Data are contained within the article or supplementary material.
