# Supplementary material for: Molecular Characterization and Phylogenetic analyses of Rotaviruses Circulating in Municipal Sewage and Sewage-Polluted River Waters in Durban Area, South Africa
Source: Food Environ Virol. 2024 Jun 24;16(3):363–79. doi: 10.1007/s12560-024-09598-z (PMC11422280; doi:10.1007/s12560-024-09598-z)
Supplement: Supplementary file 1 — Supplementary file1 (DOCX 992 KB) [file 12560_2024_9598_MOESM1_ESM.docx]

**Supplementary Fig. 1** Agarose gel results of representative Rotavirus Genotype Constellation G1 (a), G2 (b), G3 (c) G9 (d), G12 (e), P[4] (f), P[8] (g), P[6]+P[8] (h) and the NSP3 fragment (I)

M

**M**

**M**

11

10

9

8

7

6

5

4

2

1

**
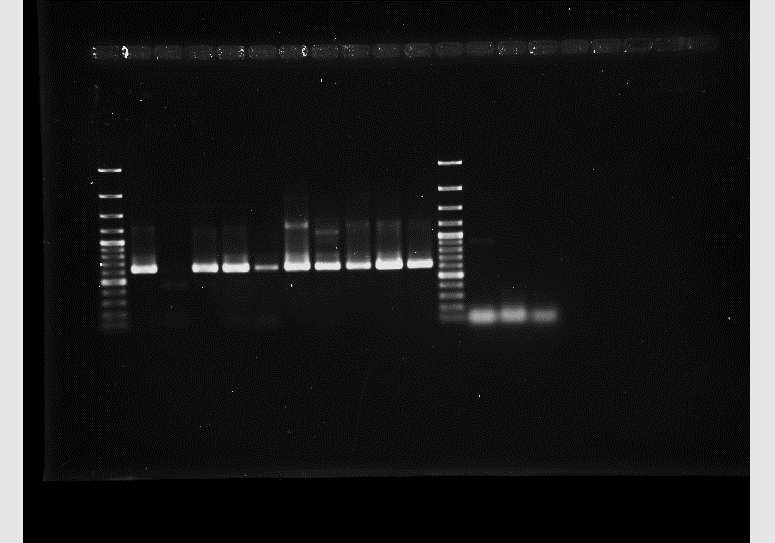
**

618bp

1. **G1** Genotype. M: 100 bp DNA ladder; Lane numbers (1-11) indicate samples; Arrow designates expected location of rotavirus G1 gene (618 bp) band

20 21 22 23 24 25 26

19

16

17

15

14

13

12

**M**

**
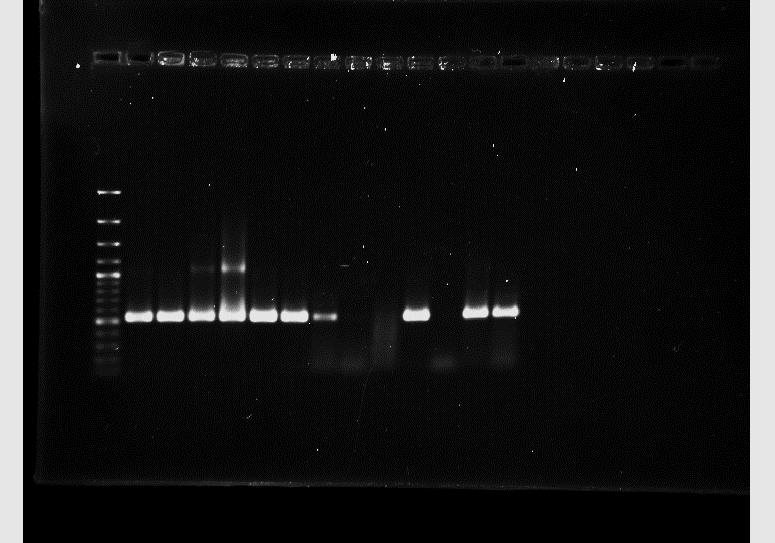
**

521bp

18

1. **G2** Genotype. M: 100 bp DNA ladder; Lane numbers (12-26) indicate samples; Arrow designates expected location of rotavirus G2 gene (521 bp) band

**M 26 27 27 28 29 30**


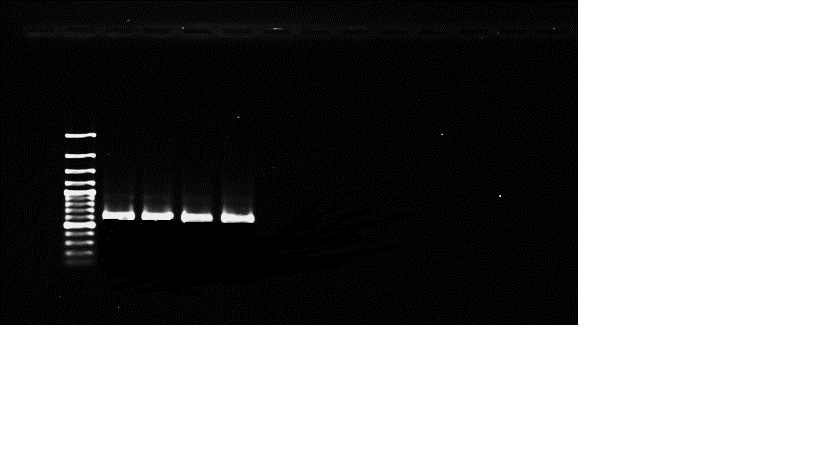


682bp

1. **G3** Genotype. M: 100 bp DNA ladder; Lane numbers (26-30) indicate samples; Arrow designates expected location of rotavirus G3 gene (682 bp) band

**M 31 32 33 34 35 36 37 38 39**

**
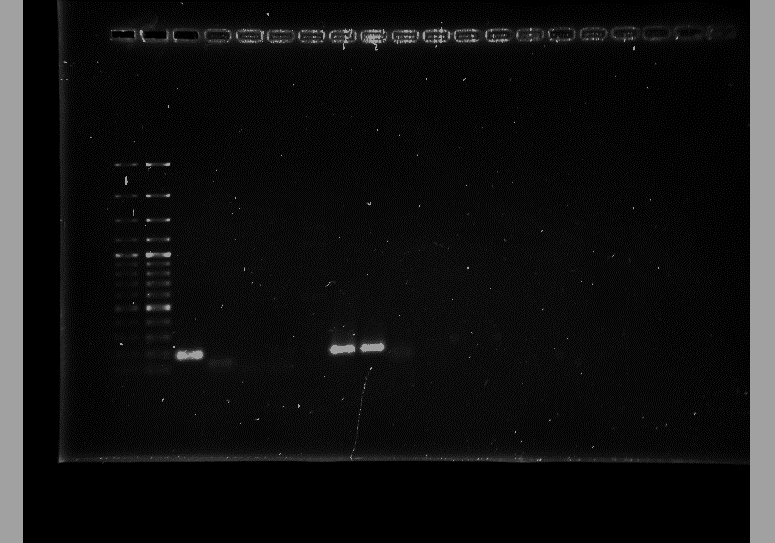
**

179bp

1. **G9** Genotype. M: 100 bp DNA ladder; Lane numbers (31-38) indicate samples; Arrow designates expected location of rotavirus G9 gene (179 bp) band

**M 39 40 41 42 43 44 45 46 47 48 49 50 51 52**

**
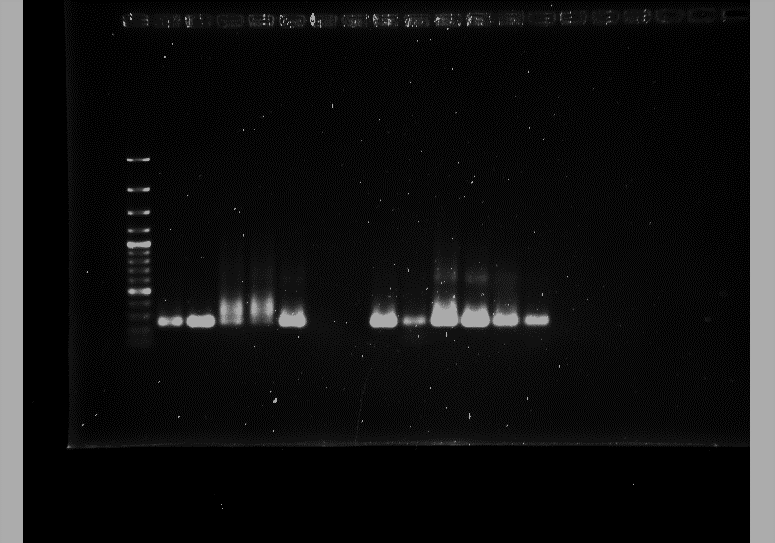
**

266bp

**e. G12** Genotype. M: 100 bp DNA ladder; Lane numbers (39-52) indicate samples; Arrow designates expected location of G12 gene (266 bp) band

**M A B C D E F G H I J K L M N O P Q R**

**
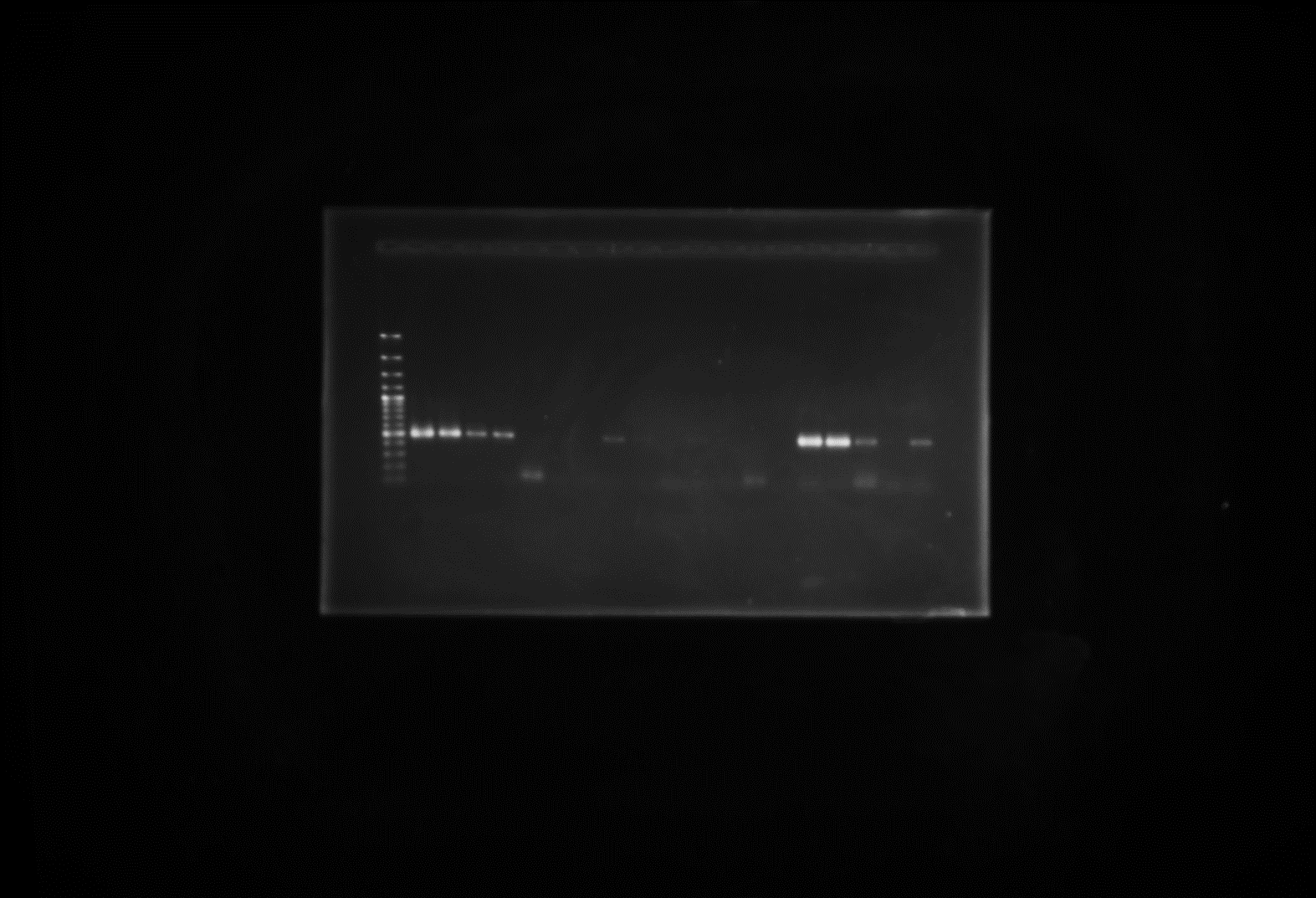
**

483bp

1. **P[4]** Genotype. M: 100 bp DNA ladder; Lanes (A-R) indicate samples; Arrow designates expected location of rotavirus P[4] gene (483 bp) band

**M 1 2 3 4 5 6 7 8 9 10 11 12 13 14**

**
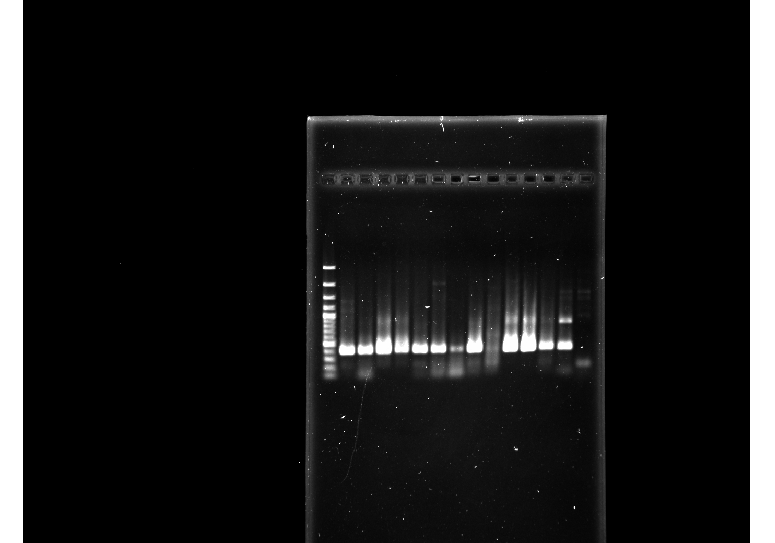
**

345bp

1. **P[8] Genotype.** M: 100 bp DNA ladder; Lane numbers (1-14) indicate samples; Arrow designates expected location of rotavirus P[8] gene (345 bp) band

**M 15 16 17 18 19 20 21 22 23 24 25 26 27 28 29 30 31 32 33**

**
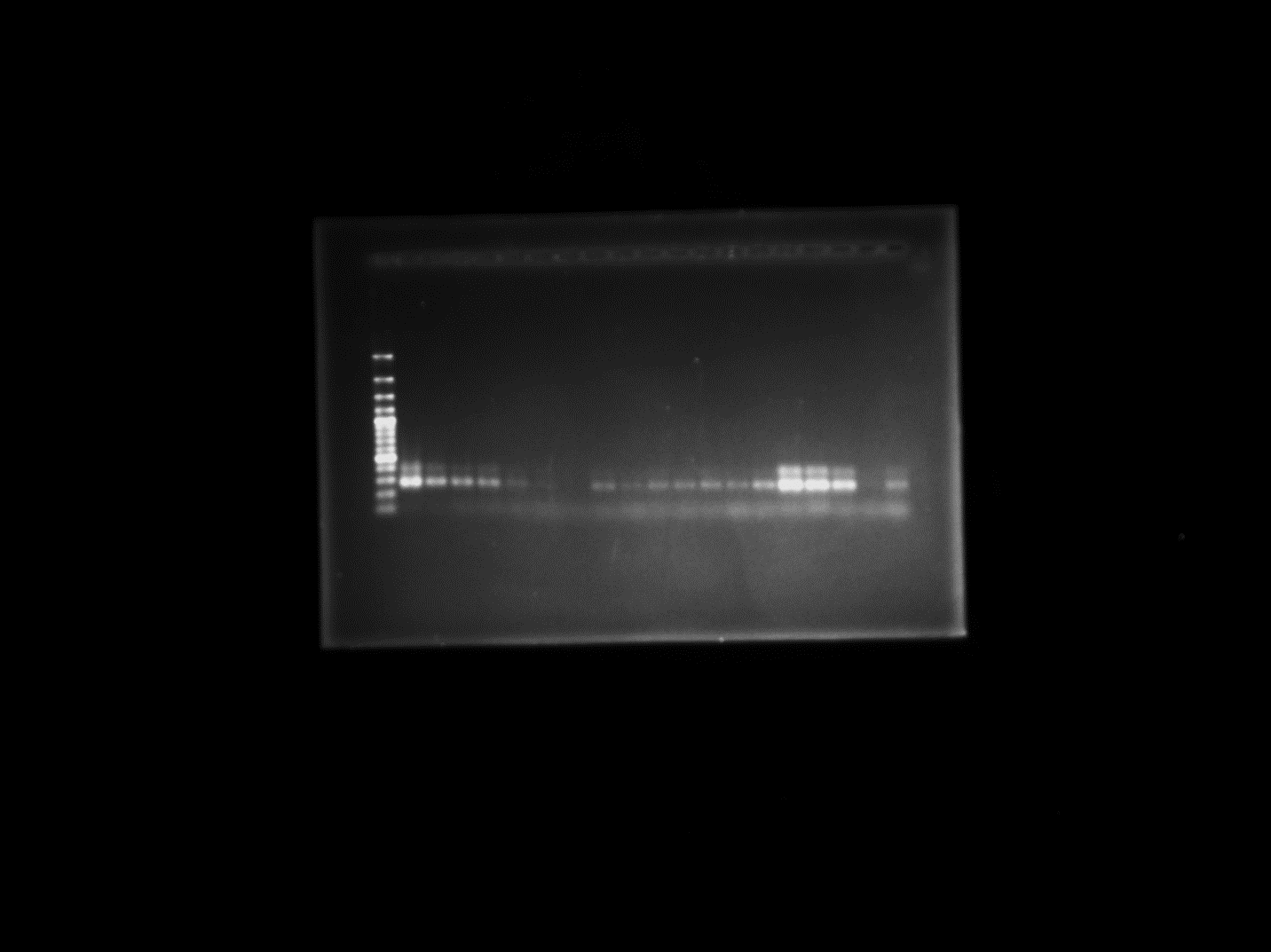
**

345bp

267bp

1. **P[6]+P[8] Genotypes.** M: 100 bp DNA ladder; Lane numbers (15-33) indicate samples; 345 bp and 267 bp bands correspond to amplified rotavirus P[6] and P[8] genes, respectively.

**Ⅿ ⅼ 2 Ꝫ 4 P N r**


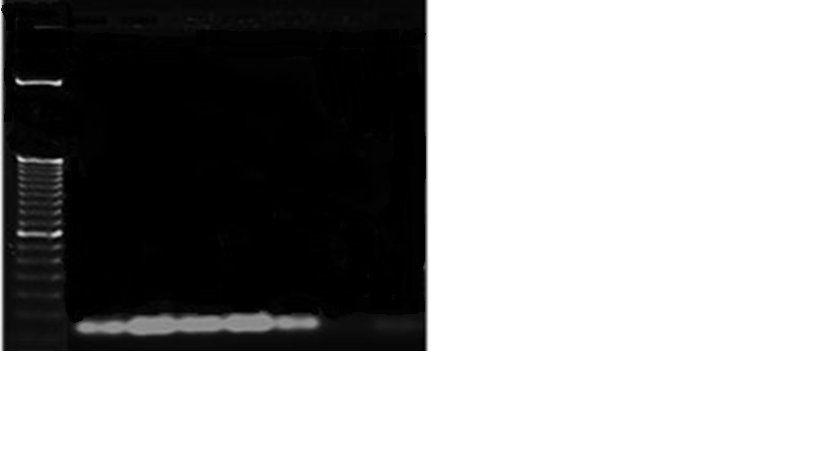


**I.** Individual PCR products stained with ethidium bromide on agarose gel. M: 50 bp DNA ladder; Numbers (1-4) indicate samples; (P) Positive control reaction; (N) negative control; (r) repeated sample. Arrow designates expected location of rotavirus NSP3 gene (87 bp) band.
