# Supplementary material for: Molecular Characterization and Phylogenetic analyses of Rotaviruses Circulating in Municipal Sewage and Sewage-Polluted River Waters in Durban Area, South Africa
Source: Food Environ Virol. 2024 Jun 24;16(3):363–79. doi: 10.1007/s12560-024-09598-z (PMC11422280; doi:10.1007/s12560-024-09598-z)
Supplement: Supplementary file 2 — Supplementary file2 (DOCX 126 KB) [file 12560_2024_9598_MOESM2_ESM.docx]

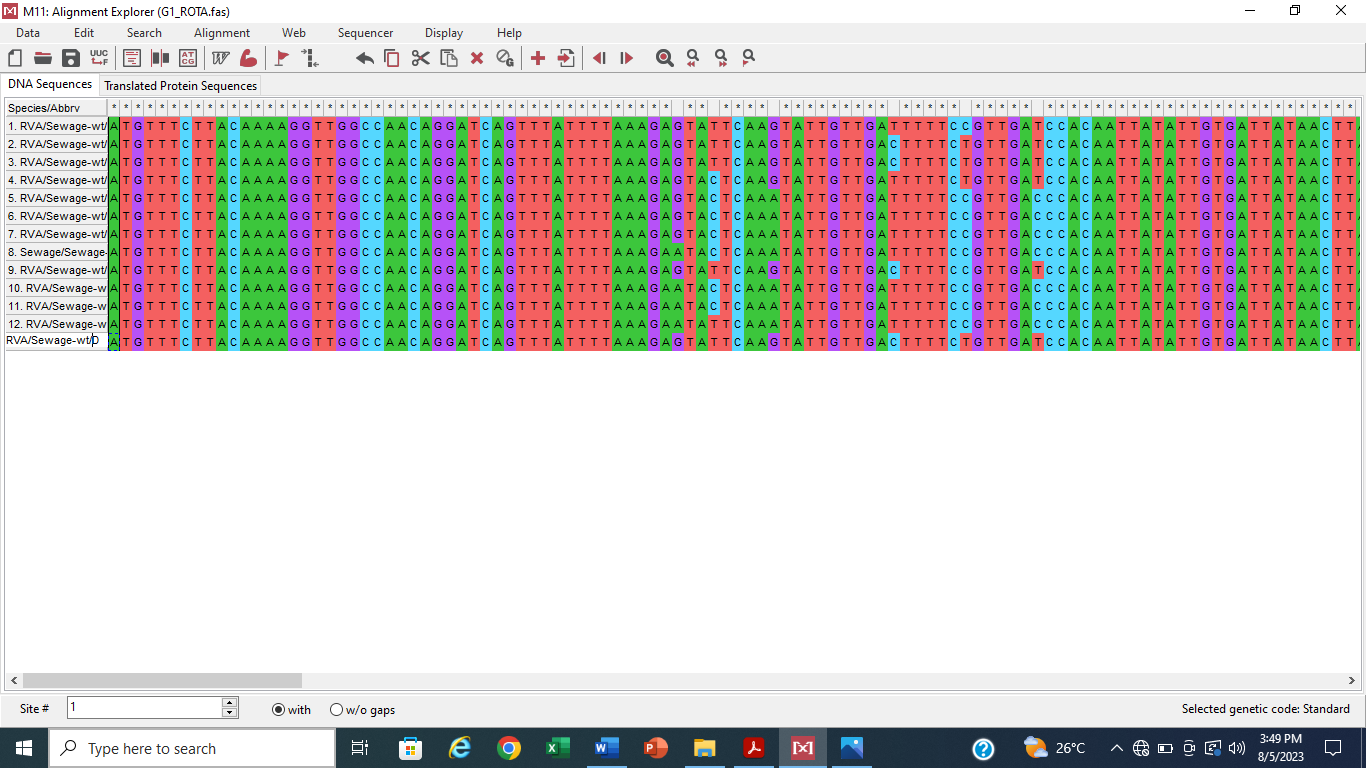


**Supplementary Fig. 2** Representative Multiple Nucleotide Sequence Alignment of Durban G1 sewage strains
